# Distinguishing autocrine and paracrine signals in hematopoietic stem cell culture using a biofunctional microcavity platform

**DOI:** 10.1038/srep31951

**Published:** 2016-08-18

**Authors:** Eike Müller, Weijia Wang, Wenlian Qiao, Martin Bornhäuser, Peter W. Zandstra, Carsten Werner, Tilo Pompe

**Affiliations:** 1 Leibniz Institute of Polymer Research Dresden, Max Bergmann Center of Biomaterials, Dresden, Germany; 2 University of Toronto, Toronto, Canada; 3 TU Dresden, University Hospital Carl Gustav Carus, Dresden, Germany; 4 Institute of Biochemistry, Universität Leipzig, Leipzig, Germany

## Abstract

Homeostasis of hematopoietic stem cells (HSC) in the mammalian bone marrow stem cell niche is regulated by signals of the local microenvironment. Besides juxtacrine, endocrine and metabolic cues, paracrine and autocrine signals are involved in controlling quiescence, proliferation and differentiation of HSC with strong implications on expansion and differentiation *ex vivo* as well as *in vivo* transplantation. Towards this aim, a cell culture analysis on a polymer microcavity carrier platform was combined with a partial least square analysis of a mechanistic model of cell proliferation. We could demonstrate the discrimination of specific autocrine and paracrine signals from soluble factors as stimulating and inhibitory effectors in hematopoietic stem and progenitor cell culture. From that we hypothesize autocrine signals to be predominantly involved in maintaining the quiescent state of HSC in single-cell niches and advocate our analysis platform as an unprecedented option for untangling convoluted signaling mechanisms in complex cell systems being it of juxtacrine, paracrine or autocrine origin.

Hematopoietic stem cells (HSCs) continuously generate mature blood cells to renew or maintain life-long hematopoiesis. The dynamic regulation of HSC number and progeny involves complex signaling mechanisms, which are strongly influenced by the local microenvironment. The urgent need for a better understanding of HSC regulation is motivated by their fundamental role in the life-long hematopoiesis and their marked regenerative potential after transplantation in clinical therapies of several diseases such as cancer or autoimmune disorders^1^,^2^. Failure of host engraftment, limited regeneration of the host hematopoietic system as well as challenges associated with *ex vivo* expansion strategies limit the success of HSC-based therapies and ask for an increased knowledge on HSC signaling^3^,^4^. In addition to juxtacrine signals from neighboring stromal cells and the extracellular matrix (ECM), a number of autocrine and paracrine signals from soluble mediator molecules have been shown to influence hematopoiesis by regulating proliferation and quiescence, as well as self-renewal and differentiation^5^,^6^,^7^. Complex interactions between these different signals are very challenging to decipher in the poorly accessible *in vivo* HSC microenvironment. Currently, a major hurdle to achieving robust *ex vivo* HSC expansion is the inability to distinguish between the autocrine and paracrine signals that govern hematopoiesis. For example signaling from VEGF via an internal autocrine loop has been shown to regulate HSC survival^8^. Notably increased expression levels of VEGF and receptors have been found in human hematopoietic tumor cell lines and there is evidence that internal and external autocrine VEGF loops regulate leukemia survival^9^,^10^. However even though other factors, e.g. FLT3L and TGF-β, have been shown to control hematopoiesis via autocrine loops as well, the autocrine or paracrine origin of most factors remains an open issue hindering current culture strategies to specifically control their supporting or adverse impact of HSC maintenance and expansion *in vitro*^11^,^12^.

A promising strategy to increase HSC expansion *ex vivo* is mimicking the HSC microenvironment by the integration of bioengineering approaches with our constantly expanding knowledge of the soluble signals involved in hematopoiesis^13^,^14^. For instance, growth factors, regulatory cytokines and chemokines essential for the tightly balanced intercellular crosstalk regulating HSC fate have been successfully identified using protein microarray technologies^15^,^16^,^17^. The presentation of these signals in an HSC culture system can be precisely controlled using biomaterial scaffolds. In fact, biomaterial scaffolds have already been designed to precisely control the presentation of growth factors (e.g., stem cell factor (SCF), stromal cell-derived factor 1 (SDF1)^18^,^19^, cell surface ligands (e.g., cadherins, Jagged1)^20^,^21^, ECM components (e.g., fibronectin (FN), heparan sulfate)^22^ and topographical features^23^,^24^ to recapitulate the bone marrow (BM) microenvironment *in vitro.* These modular toolboxes along with in-depth *in vitro* analysis provide tools to facilitate the understanding of autocrine and paracrine signaling in HSC regulation.

In the present study, we develop and use a microcavity platform to contribute to the deciphering of autocrine and paracrine signals in HSC fate regulation. Motivated by previous work of Csaszar E *et al*.^25^ we focused on the role of a large set of soluble factors with prospective impact in HSC regulation, additionally asking for the impact of juxtacrine adhesive signals from the ECM. Using silicone and hydrogel based microcavity arrays with multi-cell and single-cell sized features functionalized with FN or heparin, hematopoietic stem and progenitor cell (HSPC) proliferation and differentiation were studied *in vitro* in the context of a multiplex immunoassay analysis of cell-secreted growth factors. Based on a mechanistic model of HSC signals, a partial least squares (PLS) algorithm allowed the identification of the key players in the regulation of HSPC fate in our setting^26^,^27^. The combination of our biofunctional microcavity platform and PLS analysis introduces a novel approach that can be used to identify key molecules and their signaling mechanisms in other *in vitro* biological systems.

## Results

### Biofunctional microcavity arrays

The basic premise behind the development of our microcavity platform was that autocrine and paracrine signals, as well as juxtacrine cell-cell and cell-ECM signals, can be distinguished by comparing single-cell and multi-cell arrangements of HSPCs *in vitro*. For human HSPCs a 15 μm diameter microcavity is very well suited to establish a single cell constraint, while 40 μm diameter microcavities can already harbor up to 12 HSPCs (see next section). The cell-sized depth of the microcavities of 10 μm allows to impose spatial constraints on the cells while still enable cells to migrate and proliferate over the microstructured surface^24^,^28^. We cultured HSPCs in microcavity array scaffolds, produced from either poly(dimethyl siloxane) silicone (PDMS), 4-arm poly(ethylene oxide) (sPEG), or biohybrid sPEG-heparin hydrogels, which allowed us to vary important exogenous parameters of the HSC microenvironment, namely the local cell density and material properties of the cell culture substrate. We omitted co-culture experiments as they drastically increase the variety of possible interactions and the complexity of the system limiting by that possible conclusions. Furthermore, pure HSPC cultures were shown to be a relevant model for HSPC studies^25^,^29^.

**Figure 1:**
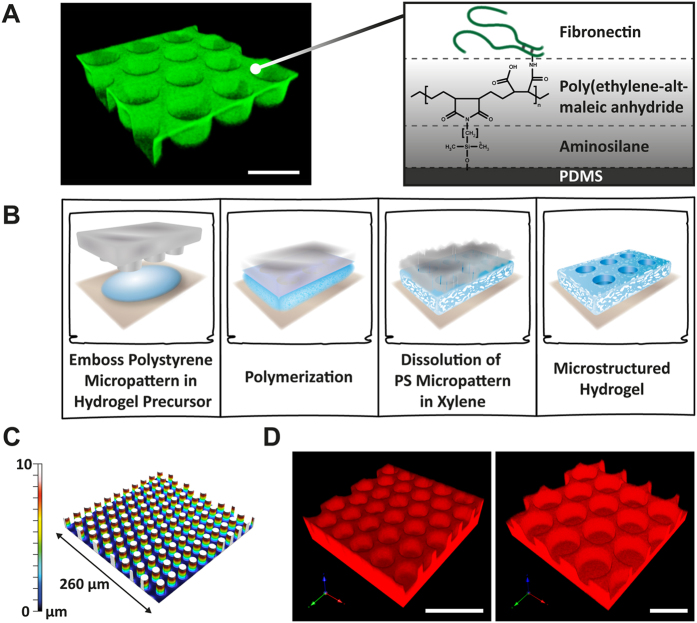
Biofunctional microcavity arrays. (**A**) Confocal laser scanning microscopy (CLSM) image of a PDMS microcavity array (Ø: 40 μm) functionalized with fluorophore-labeled FN (green) (left). Scale bar: 50 μm. Surface chemistry used for covalent immobilization of FN (right). (**B**) Schematic representation of the hydrogel micropatterning concept. (**C**) 3D image of polystyrene pillar microarray (Ø: 15 μm, h: 10 μm) derived by SAMIM and imaged by multi-pinhole microscopy. (**D**) CLSM images of sPEG-heparin hydrogel microarrays of hexagonal packed cavities with diameters of 15 μm (left) or 40 μm (right) labeled with Alexa633 fluorophore (red). Scale bars: 40 μm.

The microstructured PDMS system is well established for *ex vivo* HSC culture and we recently demonstrated it to maintain HSC functionality in mouse repopulation studies significantly better than standard cell culture^24^,^30^. PDMS microcavity arrays, with 10 μm microcavity depth and either 15 or 40 μm microcavity diameters, were produced by soft lithography. The microcavity arrays were covalently functionalized with FN, a native component of the HSC niche, using maleic anhydride copolymer thin films as a reactive coating (Fig. 1A)^23^. These scaffolds provide adhesion ligands for integrin mediated interactions, exhibit a high stiffness (shear modulus 2 MPa) and are non-penetrable for small growth factors and nutrition factors.

In contrast, the hydrogel microcavity system is based on end-functionalized sPEG crosslinked with heparin as a functional glycosaminoglycan^31^,^32^. Due to the physicochemical properties of the hydrogel scaffolds, namely highly hydrophilic and fragile, a new technology applying sacrificial polymer microstructures, patterned via solvent-assisted micromolding (SAMIM), was used to fabricate micro-structured hydrogel scaffolds with dense microcavities (Fig. 1B–D). Thereby we could create 10 μm deep, densely hexagonal packed microcavities as small as 15 μm with 3 μm thick walls between the microcavities. These hydrogel microcavity arrays were made of either pure sPEG (sPEG-sPEG) or sPEG crosslinked with heparin (sPEG-HEP). The very soft hydrogel microstructures (storage modulus 1–8 kPa) retained their shape even after several weeks under cell culture conditions. The heparin feature of the hydrogel scaffold was optionally used for covalent functionalization with adhesion-mediating RGD peptides. Furthermore, the heparin component is known to regulate the binding and release of various cytokines^33^,^34^.

### *In vitro* culture of CD34+ HSPCs on top of microcavity arrays

To examine the effects of spatial constraint and material properties on HSPC expansion, 3·10^4^ GCSF mobilized human CD34+ HSPCs, isolated from the peripheral blood of healthy donors (after obtained informed consent), were seeded on top of microcavity arrays made from either hydrogels (sPEG-HEP and sPEG-sPEG) or PDMS. Tissue culture plastic (TCP) substrates served as controls. The cultures were supplemented with SCF, TPO and FLT3L at 10 ng·mL^−1^, the minimum concentration known to maintain or stimulate mild proliferation of HSPC over 7 days^24^. The low cytokine concentrations were chosen to avoid a very high proliferation reported at higher concentrations which could override the subtle influences exerted by our microarray’s spatial constraint and material properties^24^,^35^.

**Figure 2:**
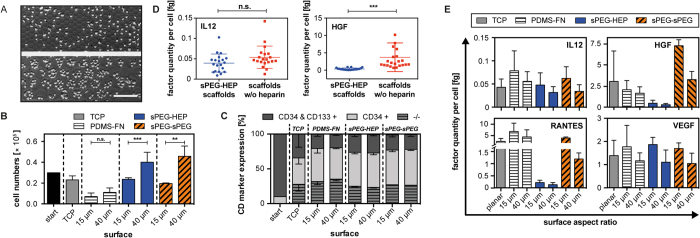
HSPC expansion and growth factor expression of CD34+ HSPCs cultured on top of microcavity arrays. (**A**) HSPCs cultivated for 2 days on top of hydrogel microcavity arrays with cavities of 15 (top) and 40 μm (bottom) diameters. The phase contrast microscopy images prove the 15 μm microcavities to be single-cell cavities, while the 40 μm microcavities contain multiple cells (up to 12) (scale bar: 80 μm). (**B**) Overall cell number of HSPCs cultured for 1 week on top of TCP and microcavity arrays consisting of FN functionalized PDMS, sPEG-heparin and sPEG hydrogel scaffolds, grown at cytokine background level of 10 ng·mL^−1^. All data are presented as mean ± SD from one donor with n = 2–3 microcavity samples per donor. Statistical significance is denoted as follows: *P < 0.05, **P < 0.01, ***P < 0.001. (**C**) Percentage of cells expressing CD34 and CD133 in the total cell population. All data are presented as mean ± SD from one donor with n = 2–3 samples per condition. (**D**) Plot shows the influence of heparin content of the microcavity arrays on growth factor levels within the cell culture supernatants exemplarily shown for sequestered (HGF) and non-sequestered (IL12) cytokines. Results are presented as mean ± SD from 3 donors with n = 2–3 microcavity arrays per condition and donor. (**E**) Growth factor levels were measured in the supernatant of day 7 of culture using multiplex analysis. Plot shows measured growth factor levels normalized with the final cell number. Exemplary factors are presented as mean ± SD from either 1 donor (sPEG-sPEG), 2 donors (PDMS-FN), or 4 donors (sPEG-HEP/TCP) with n = 2–3 samples per condition and donor.

Over a 7 day culture period, HPSC number declined on all surfaces except for 40 μm diameter hydrogel microcavities (Fig. 2B and SI, Fig. S1C). The lowest HSPC expansion was observed on PDMS-FN scaffolds, regardless of microcavity diameter. The presence of heparin in hydrogel scaffolds had no apparent effect on HPSC expansion. Notably, although cell growth was affected by the scaffold material, the impact of microcavity size was similar for all three materials. Cell numbers were always significantly lower on 15 μm microcavity arrays containing individual cells than on multi-cell 40 μm microcavity arrays. Figure 2A proves the characteristics of the 15 μm (single-cell) microcavities to harbor only single cells, while 40 μm (multi-cell) microcavities contain up to 12 cells.

While increasing the concentration of supplementary cytokines (SCF, TPO, FLT3L) in the culture medium beyond physiological concentrations (30 ng·mL^−1^) did yield cytokine-driven HSPC proliferation, it did not appear to influence the differential impact of microcavity size and material properties on cell expansion (SI, Fig. S1C). Similarly, decreasing the initial cell number to 1.5·10^4^ cells per surface did not influence microcavity-dependent cell expansion (SI, Fig. S1A). Based on these observations, we conclude that the total microcavity surface was large enough that we never observed a complete occupation of all the microcavities on a scaffold. The final cell number was always lower than the maximum cell number capacity of the microcavity arrays (i.e. 3.3·10^5^ for 15 μm microcavity arrays and 7.2·10^5^ for 40 μm microcavity arrays).

Whereas the scaffold material and microcavity diameter were found to affect proliferation, the expression frequency of stem cell surface markers CD133 and CD34 was similar in all our culture conditions with 64 to 76% CD34+ HSPCs (Fig. 2C). Thus, the highest total number of CD133+/CD34+ HSPCs was achieved on the hydrogel microcavity arrays after a 7-day culture period (Fig. 2B,C).

The use of CD34 and CD133 as markers of a HSPC subset with enriched repopulating capacity is motivated by its known clinical relevance and previous studies^36^,^37^. It has to be kept in mind, that the CD34+/CD133+ subpopulation is not a pure HSC population, but only enriched for primitive blood progenitors. However, only xenotransplantation studies would allow for more relevant insights in the subpopulation characteristics, which is beyond the scope of this screening study. Furthermore, the impact of spatial constraints of PDMS-FN microarrays on HSPC in the murine model have been previously described by us including clonogenicty, proliferation, and *in vivo* transplantation demonstrating the relevance of HSPC proliferation and surface marker studies^30^.

Although HSPC proliferated differently in respect to material parameters (Fig. 2E), the frequency different subpopulations derived from the starting CD34+ selected cells appeared to be consistent between different treatment conditions (Fig. 2B,C). These results suggest that the signals controlled by the microarray configurations mainly impact HSPC cell division rates and not lineage bias.

To determine the regulatory signals involved in HSPC cell-cell communication via soluble cell-secreted factors we analyzed a large set of secreted signaling molecules after 7 days of culture. Using multiplex immunoassays, we detected significant levels of the following factors involved in HSPC fate decisions: IL8^38^, IL12^39^, ANG-2^40^,^41^, GM-CSF^42^, HGF^43^,^44^, FST^45^, MIP-1β^25^, PDGF-AB/BB^45^, PECAM-1^46^, RANTES^47^, TGF-β1^48^, and VEGF^8^ (SI, Fig. S2). Factor levels were normalized to corresponding cell numbers at day 7 of culture. The highest factor concentrations per cell were measured for ANG-2, HGF, RANTES, TGF-β1, and VEGF (SI, Fig. S3). We observed that the secreted factor quantities (per cell) had a trend to be higher in the 15 μm microcavity arrays compared to the 40 μm microcavity arrays. This effect was found to be independent of scaffold material and secreted factor (Fig. 2E and SI, Fig. S3). Furthermore, we discovered that general levels of most of the secreted factors were related to the heparin content of the scaffold material irrespective of cavity size, while a minor fraction of other factors were not, see Fig. 2D and Fig. S5. With the exception of IL12, ANG-2, TGF-β1 and VEGF, measured factor levels per cell were always lower on microcavity arrays containing heparin in comparison to scaffolds without heparin (including TCP controls).

Overall, the above results suggest a complex interplay between impacts of microcavity size, scaffold material, and secreted factors in HSPC maintenance.

### A mechanistic approach to reveal key parameters of *in vitro* HSPC culture

While it is clear that there is a relationship between microcavity size, scaffold material, and secreted signals on HSPC expansion, our results are difficult to interpret due to the limited information available about the synergistic interplay of the detected factors, the concentration levels for bioactivity, degradation rate via receptor internalization, and enzymes and their role in HSPC fate decisions.

**Figure 3:**
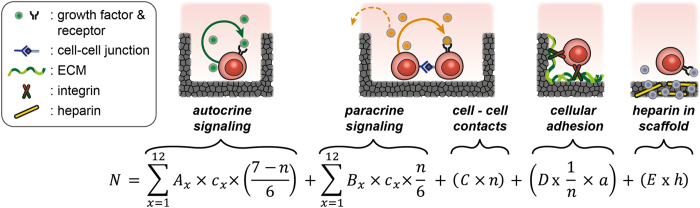
Mechanistic signaling model of HSPC *in vitro* culture. The linear model depicting the correlation between HSPC number, autocrine and paracrine signaling activity, cell-cell contacts, cell-ECM adhesion and heparin content of the microcavity arrays is illustrated. N: cell numbers; c_x_: concentrations of factor x; A_x_: stimulative or inhibitory effect of factor x by autocrine signaling, n: cell-cell contacts (15 μm cavities: 1, 40 μm cavities: 3.6, planar: 6); B_x_: stimulative or inhibitory influence of factor x by paracrine signaling, C: factor independent influence of cell - cell contacts; D: factor independent influence of adhesion to scaffold; a: material specific adhesion parameter (PDMS-FN: 1, TCP: 0.5, PEG-HEP&PEG-PEG: 0); E: stimulative or inhibitory effect of heparin; h: heparin content in the scaffold: (PEG-HEP: 1, PEG-PEG&PDMS-FN&TCP: 0).

In order to reveal essential information on HSPC signaling affected by environmental parameters in the context of HSC maintenance and proliferation, we setup a simple mechanistic model describing HSPC expansion in terms of the interplay between autocrine, paracrine and juxtacrine signals mimicked by our microcavity platform (Fig. 3). This model comprises the following components: (1) We hypothesized that the influence of autocrine and paracrine signaling activity of secreted factors can be estimated by their inhibitory or stimulatory effects on cell number, while autocrine and paracrine activity is attenuated or amplified with increasing microcavity size. Hence, we considered autocrine signaling to be strongest in microcavities hosting individual cells, as they do not have any adjacent cells. In contrast, the level of paracrine signaling is considered to increase with higher numbers of cells contained in a microcavity. (2) Juxtacrine signals between cells were defined as a cell-cell contact parameter, calculated on the basis of hexagonal packing and an average occupation of the large cavities by 9 of 12 maximal possible cells. Juxtacrine signals from precoated or cell-secreted adhesion ligands were treated as the same parameter in a reciprocal manner given that cells are expected to have more cell-ECM contacts in small microcavities allowing for bottom and side contacts. Cell-ECM interactions were considered strong for FN-coated PDMS, intermediate for adhesive TCP surfaces and negligible for PEG-containing hydrogels. Lastly, heparin content of the scaffold was included as a sequestering parameter implying that secreted factors in the medium with heparin-affinity may be absorbed from the medium into the bulk volume of the hydrogel scaffold^33^,^34^. We deliberately limited the model to linear influences of the described signals. High-order terms and interdependencies would overstretch the validity and possible conclusions of the model.

The highly complex parameter set based on the experimental data and the introduced model was evaluated using a PLS algorithm, as such methods have been found useful to analyze high-dimensional data, especially if variables *n* are much larger than measured parameters *p*. Within the PLS algorithm, we applied a nonlinear iterative partial least squares fitting algorithm (NIPALS) to calculate the coefficient values of our mechanistic model and the impact of the *x* variables on their explanation of the *y* response, by calculating the variable importance (VIP) value of each variable^27^,^49^,^50^,^51^.

**Figure 4:**
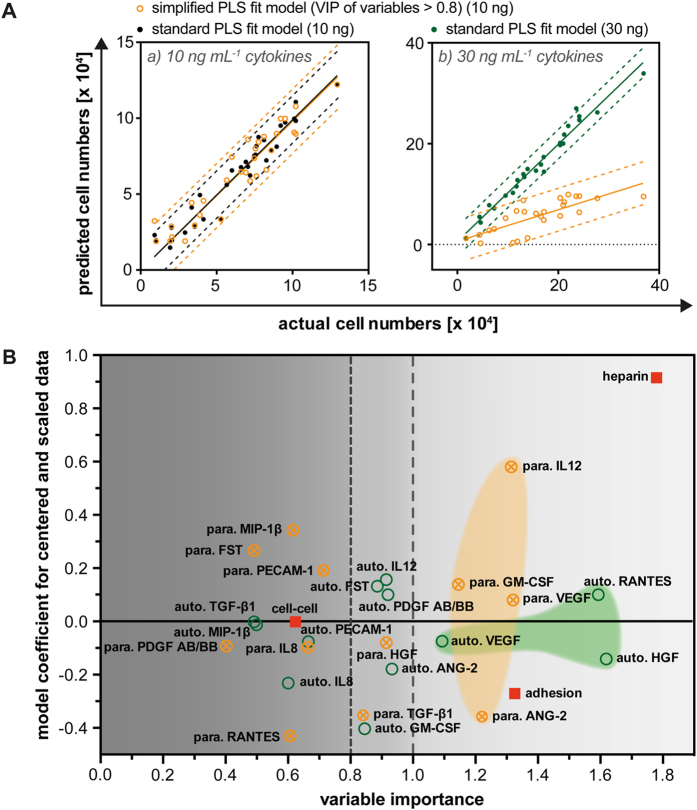
Partial least squares analysis of the mechanistic model. (**A**) Model coefficients and corresponding VIP values were calculated for the data obtained from the 7 day cultures of HSPCs on top of TCP and PDMS-FN, PEG-HEP, or PEG-PEG microcavity arrays using 10 ng·mL^−1^ background cytokines in the medium. The dimensions of the model were reduced by deleting all variables with variable importance in projection (VIP) scores < 0.8. The simplified model was compared to the standard PLS fit model concerning the obtained cell numbers. The simplified model with fitted coefficients was validated by application to the set of data obtained from similar HSPC experiments, but at 30 ng background cytokine levels. Dashed lines of both graphs illustrate the 90% prediction range. (**B**) Plot shows the calculated model coefficients for centered and scaled data as a function of VIP. Coefficients with small values, close to 0, and VIP scores < 0.8 have no significant influence on the explanation of the *y*-response. The abbreviations ‘auto.’ and ‘para.’ represent autocrine and paracrine contributions in the model, respectively.

As shown in Fig. 4A the predicted cell numbers determined from the PLS calculated model coefficients fit almost all the experimentally obtained cell numbers. Reducing the dimension of the formula to variables having VIP ≥ 0.8, typically used in PLS analysis as the limit in significance, we again could nicely predict the cell numbers with high accuracy (R^2^ = 0.95)^52^. To validate the VIP optimized model, we applied the simplified formula to predict the cell number results obtained in a different experiment supplemented with 30 ng·mL^−1^ background cytokines (SCF, TPO, FLT3L) instead of 10 ng·mL^−1^. Although we obtained a linear relationship between the predicted and measured cell numbers again (Fig. 4A (right)), the accuracy was lower (R^2^ = 0.61). The slope of the linear regression calculated by the 10 ng·mL^−1^ model was about 3-fold lower leading to a 3-fold underestimation of cell number. This indicates that the cytokine background of SCF, TPO and FLT3L scales proliferative activity of HSPC independent of specific parameters of the microcavity and material environment.

To reveal the level and significance of stimulatory and inhibitory effects that each factor under investigation has on HSPC cell number, we plotted their model coefficients against their corresponding VIP values (Fig. 4B). From this, we observed that heparin plays the most important role on HSPC growth in our system because it has the highest positive coefficient value coinciding with the highest VIP score (VIP = 1.8). Also significant (VIP = 1.35), juxtacrine cell-ECM interactions (adhesion) had a negative coefficient value. Analyzing significant autocrine and paracrine signals we found 6 factors with paracrine and 8 factors with autocrine activity (VIP > 0.8), with both signaling pathways having a balanced number of positive and negative factors. While for 5 factors (IL12, ANG-2, GM-CSF, HGF VEGF) both autocrine and paracrine signals were found, one has to recognize that we never found similar autocrine and paracrine activities (similar VIP and coefficients) for one factor. This points to distinct autocrine or paracrine signaling for the found factors. Focusing on the most important variables (VIP > 1.1) we recognized that there is a separation of the factors involved in autocrine (HGF, RANTES) and paracrine signaling (VEGF, IL12, ANG-2, GM-CSF). Autocrine signals appeared to be more significant, however with smaller coefficient values, than paracrine signals. This observation is schematically illustrated via the green and orange marked areas in Fig. 4B.

**Figure 5:**
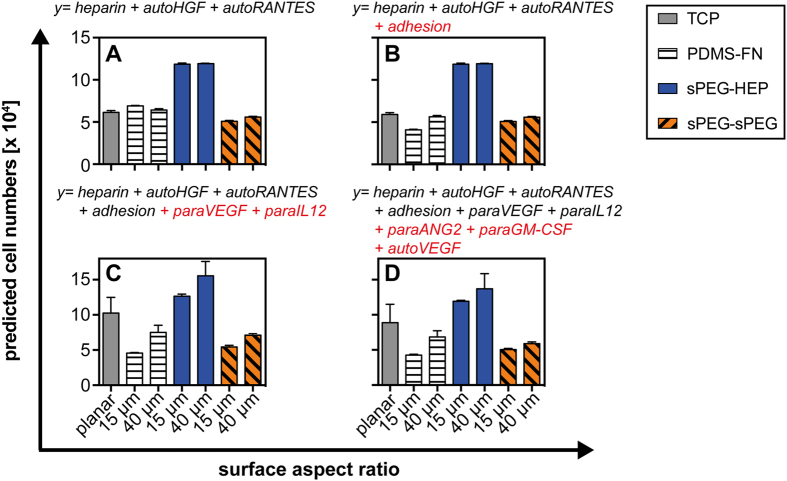
Minimized models applying PLS coefficient values. To reveal the impact of autocrine and paracrine signaling, as well as material characteristics, we minimized the PLS derived model (VIP > 1.1) and systematically rebuilt it starting with the factors that had the highest VIP scores. Plots (**A–D**) show the predicted cell numbers calculated by the stepwise model extension with factors added with respect to decreasing VIP scores with (**A**) VIP > 1.6, (**B,C**) VIP > 1.3 and (D) VIP > 1.1.

The latter finding motivated us to minimize the original model in order to reveal key parameters in HSPC regulation. We systematically rebuilt our original model using the PLS derived coefficients. We started with the three factors (heparin, autoHGF, and autoRANTES) that had the highest VIP scores (VIP > 1.6) and then in stepwise fashion added the 3 factors (1. adhesion, 2. paraVEGF+ paraIL12, and 3. paraANG2 + paraGM-CSF) with the next-highest VIP values (1.6 > VIP > 1.1) one factor at a time (Fig. 5).

By applying the model coefficients for heparin, autoHGF and autoRANTES, (VIP > 1.5) we observed that material characteristics, namely heparin content, not microcavity size, impacted cell number (Fig. 5A). The low influence of the autocrine signaling of HGF and RANTES on the microcavity size dependent cell growth (small coefficients in the model, see Fig. 4) is only seen on sPEG-sPEG, not on sPEG-HEP and PDMS-FN (Fig. 5A). By extending the model to include the coefficient for adhesion (VIP = 1.33), a microcavity size dependence was observed, but only on PDMS-FN (Fig. 5B). A significant impact of microcavity size on cell number was observed in all materials upon introducing model coefficients for paracrine signaling factors VEGF and IL12 (VIP = 1.32 and VIP = 1.31, respectively) (Fig. 5C). Implementation of the remaining factors (1.1 < VIP < 1.3) resulted in a slight increase in the accuracy of predicted cell numbers but not in any significant refinement of the general trends (Fig. 5D).

## Discussion

By applying a platform of biofunctional microcavity arrays, we revealed key parameters in autocrine, paracrine and juxtacrine signaling of HSPCs. Our results reinforce the concept of using spatially defined niches, reflecting the environment inside the BM, to control stem cell quiescence and expansion over long time periods. We demonstrated that our microcavity array platform can be used to control microenvironmental signals that affect HSPC expansion. The HSPC expansion data were used to validate a newly established mechanistic model by a PLS approach to identify key parameters in the convoluted signaling system of HSPC regulation. By that we found autocrine signals in single-cell niches to be critical in HSPC quiescence. Finally, the sequestering of heparin-binding cytokines by the hydrogel scaffold was identified as a new tool to control HSPC maintenance.

A novel feature of our microcavity platform is that it enables the separation of autocrine and paracrine signaling mechanisms of certain mediators of *in vitro* HSPC culture. Autocrine signals from RANTES and HGF were shown to have a small, but significant, effect on cell growth, while their paracrine signals were shown to be not relevant. The found model coefficients agree to reports on HSPC proliferation showing a supporting function of RANTES (*in vitro* exposure of HSPCs to RANTES yields increased levels of HSPC after transplantation *in vivo*) and hindering influence of HGF (diminished proliferation during *in vitro* HSPC expansion)^25^,^47^. Their small impact only on non-adhesive and non-factor binding sPEG-sPEG hydrogels is related to the strong factor depletion of RANTES and HGF by heparin in sPEG-HEP hydrogels and the juxtacrine signals on adhesive PDMS-FN microcavity arrays^24^,^53^. It is expected that autocrine signaling is strongly affected by factor depletion via heparin in small microcavities due to the large nearby material surface area. This feature nicely fits the low abundance of HGF and RANTES in HSPC cultures on heparin-containing scaffolds correlating to their heparin affinity known from literature^54^,^55^. We believe that RANTES and HGF can be treated as examples of the effect of heparin-driven depletion of autocrine signals. Other factors involved in hematopoiesis or HSPC fate decisions^16^,^17^,^40^,^45^,^56^,^57^, not monitored in our experiments, are suggested to follow the same behavior concerning enhanced depletion of heparin-binding autocrine mediators. This assumption is supported by the high coefficient and VIP of the heparin parameter. Although we currently cannot proof this interpretation by measuring the wealth of other suggested soluble mediators in HSPC signaling, we would propose the heparin parameter to indirectly cover the non-measured autocrine signals in our model.

On the other hand, paracrine signaling cytokines IL12 and ANG-2, both not affected by adsorption to heparin in our measurements, contribute to a strong material independent microcavity effect. Again, the found model coefficients agree to reported effects in HSPC proliferation with a supporting function of IL12, mostly in a synergistic manner with other factors such as SCF, and a hindering influence of ANG-2 (reduced number of functional HSC after *in vitro* exposure)^7^,^40^,^56^. Paracrine signaling should be more favored in large microcavities due to the close association of neighboring cells. Similarly, heparin depletion of paracrine signaling factors should only have a minor effect because of the smaller ratio of cells to material surface area. In agreement, our model predicted a size-dependent effect from IL12 and ANG-2 signaling, with higher cell numbers on the large microcavities.

These arguments on autocrine and paracrine signals allow us to conclude that in single-cell microcavities autocrine signaling is the prevalent pathway. The occurrence of single-cell HSC niches was reported from *in vivo* studies^6^,^58^,^59^. Furthermore, we previously demonstrated that *in vitro* culture of HSPCs on top of microcavity arrays hosting individual cells supports stem cell functionality^24^,^30^. We hypothesize that the more quiescent state of HSCs within the spatially constraint single-cell niche could be the result of increased autocrine and decreased paracrine signaling.

Remarkably, the presence of heparin in the cell culture scaffolds was found to be the most important variable in the PLS analysis, with the highest VIP and most positive coefficient value of our model. Systematically rebuilding our model by stepwise extension with variables with decreasing VIP score, we revealed that the influence of heparin content is not related to microcavity size. As we measured lower factor levels in the cell culture supernatants of heparin containing hydrogels, we expect heparin to support HSPC maintenance and proliferation by binding and sequestering a multitude of secreted signaling molecules. Notably, with the exception of ANG-2, all analyzed factors have reported heparin affinity^46^,^54^,^55^,^60^,^61^,^62^,^63^,^64^,^65^,^66^,^67^,^68^. We recently demonstrated that factor depletion promotes HSPC growth, supporting our findings on altered HSPC cycling in the presence of heparin containing hydrogels^25^. Finally, we recognize that our model could not fully reconstruct the experimental data on HSPC proliferation, see Fig. 2B, in particular for the sPEG hydrogels without heparin content. We assume that, in addition to the sequestering effect of heparin, other factors, not measured in our assays, can be similarly sequestered by hydrophobic interactions with maleimide groups also present in the pure sPEG hydrogels. This effect is currently not included in the model. However, it could account for the lower total cell number in the modeled data, see Fig. 5D.

Juxtacrine signals via cell-ECM adhesions were found to drastically reduce cycling of HSPCs corresponding to previous reports^24^. However, cell-ECM adhesion could only partially explain the impact of microcavity size on HPSC expansion. Autocrine and paracrine signals act on HSPC cycling, too. Furthermore, our PLS analysis indicated that, in contrast to previous reports, a minor impact of direct cell-cell interactions of HSPCs exists^20^,^21^,^24^,^69^.

Furthermore we also made an observation regarding material stiffness and HSPC expansion. The application of our new solvent-assisted micropatterning approach allowed us to demold very soft and fragile hydrogel microstructures in addition to our standard PDMS microstructures. As both hydrogels and PDMS exhibit distinct mechanical properties, we also tested the implementation of material stiffness into our mechanistic model. In contrast to recent reports, we did not observe that material stiffness (VIP < 0.8) affected HSPC expansion^70^.

In summary, our findings suggest that micron-sized spatial constraints allow for creating HSC microenvironments with gradated functionality and impact on cell fate decisions. Functionalized microcavity arrays together with multiplex analysis and statistical analysis were proven useful to decipher specific signaling pathways of the dynamic regulation of HSPC. The introduced methodology can be applied to multiple related studies centering around the microenvironmental regulation of stem cells.

## Methods

### Fabrication of PDMS microcavity arrays

Fabrication of PDMS microcavity arrays was performed as described previously^23^. In brief, silicon masters were created with symmetric arrays of pillars, possessing a diameter of 15 or 40 μm, a height of 10 μm, and an intercolumniation of 3 μm using photolithographic etching (GeSiM, Rossendorf, Germany). They were fluorosilanized via gas phase with (heptadecafluoro-1,1,2,2-tetrahydrodecyl)-dimethylchlorosilane (ABCR) prior to PDMS casting. PDMS microcavity arrays were created by replication of thin films of PDMS (Sylgard 184 silicone elastomer kit, Dow Corning, Wiesbaden, Germany) from the microstructured silicon wafers using a ratio of 10:1 for curing agent and prepolymer and curing at 120 °C for 2 h.

After curing, PDMS replicas were carefully peeled off the silicon master and unreacted prepolymer and curing agent were removed by swelling in heptane (Merck, Germany) for 24 h. Afterwards heptane was entirely removed by storage for 24 h in a vacuum oven at 60 °C, PDMS microcavity arrays were punched with a 11 mm punch press and attached on top of cleaned glass slides (Ø 11 mm). Subsequently the PDMS surfaces were treated with low pressure oxygen plasma (PDC-002, Harrick Plasma, NY, USA) for 60 s and incubated in a solution of 20 mM

3-aminopropyl-triethoxy-silane (ABCR) dissolved in ethanol (Sigma-Aldrich) for 2 h. After intense washing in ethanol and subsequent annealing for 1 h at 120 °C films of poly(ethylene-*alt*-maleic anhydride) (PEMA) (MW 125000, Sigma-Aldrich) were produced by adding 40 μL of a 0.3_wt_% solution of PEMA dissolved in ethanol on the amino-functionalized surface. PEMA was covalently linked to the surface by annealing at 120 °C for 2 h. Afterwards excess PEMA was removed by autoclaving in MilliQ for 20 min at 120 °C (autoclave system, *DX23*, Systec, Germany).

To covalently bind FN, the anhydride moieties on the surface of PEMA-coated PDMS microcavities were regenerated at 120 °C for 2 h and the microcavity arrays were incubated in 40 μg·mL^−1^ FN (Roche, Germany) dissolved in phosphate buffered saline (PBS) (Sigma-Aldrich) for 1 h. Finally, surfaces were washed in PBS and CellGro^®^ SCGM medium (CellGenix, Germany) prior to cell culture.

### Fabrication of sPEG microcavity arrays

At first, sacrificial polystyrene (PS) microstructures were fabricated via SAMIM^71^. Therefore, PDMS microstructures, embedded in polycarbonate holders (GeSiM), were molded (10:1 curing agent to prepolymer) from the microstructured silicon masters at 60 °C for 4 h and incorporated in a semiautomatic micro-contact-printing system (μ-CP 3.0, GeSiM). The PDMS membrane was wetted with 26 μL of ethylacetate (Sigma-Aldrich), and immediately pressed into a flat PS sheet with a thickness of 400 μm (Evergreen Scale Models, Woodinville, WA, USA). After 15 min at 25 °C the PDMS pattern was carefully removed leaving a microstructured PS surface. The topography and dimensions of the patterned PS surfaces were analyzed via multi-pinhole confocal microscopy utilizing a μsurf explorer (NanoFocus AG, Oberhausen, Germany) equipped with a 60x objective (MoPlan APO 60x/0.9 Olympus Deutschland GmbH, Hamburg, Germany), providing a z-resolution of 2 nm and an x-y-resolution of 500 nm. Illustrated 3D maps were generated using μSoft analysis software (NanoFocus AG).

Next glass coverslips were modified with a reactive maleic anhydride copolymer coating as described in detail by Pompe *et al*.^72^. Coverslips with a diameter of 11 mm (Menzel, Germany) were cleaned, oxidized in a solution of hydrogen peroxide (Merck, Darmstadt, Germany), ammonia (Fisher Scientific GmbH, Germany) and deionized water (ratio of 1:1:5 (v/v/v)), and incubated for 2 h in a solution of 20 mM 3-aminopropyl-triethoxy-silane (ABCR, Karlsruhe, Germany) in isopropanol (Sigma-Aldrich, MO, USA) and deionized water (ratio of 9:1 (v/v)). The amino-functionalized carriers were rinsed in isopropanol and annealed at 120 °C for 1 h. Thin films of poly(ethylene-*alt*-maleic anhydride) (PEMA) (MW 125000, Sigma-Aldrich) were produced by spincoating (*RC5*, Suess Microtec, Germany) 30 μL of a 0.3_wt_% solution of PEMA dissolved in acetone and tetrahydrofuran (THF) (1:2 (v/v)) (both Sigma-Aldrich). The coverslips were treated at 120 °C for 2 h to covalently link the polymer film to the carrier. Excess polymer was removed by washing in acetone. Prior to immobilization of sPEG-heparin hydrogels, reactive anhydride groups of PEMA coated glass slides were created by treating for 2 h at 120 °C and in the following incubated in a solution of 100 μg·mL^-1^ N-(2-Aminoethyl)maleimide (Sigma-Aldrich) dissolved in MilliQ. After 30 min of incubation the surfaces were rinsed with MilliQ and dried. Hydrogels were formed via maleimide-thiol Michael-type addition using a polymer content of 5 wt-% with a molar ratio of sPEG to heparin of approximately 2:1. Heparin (Merck-Millipore, Darmstadt, Germany) functionalized with 8 maleimide units per molecule (synthesized by M. Tsurkan, IPF Dresden) was dissolved in PBS and ultrasonicated for 1 min in ice-cooled water. At the same time thiol end-functionalized 4-arm sPEG was dissolved in PBS by ultrasonication and pH was adjusted to pH 5. In the case of pure sPEG hydrogels, maleimide end-functionalized 4-arm sPEG was used instead of heparin. A volume of 7.5 μL of each thiol functionalized sPEG and heparin or maleimide functionalized sPEG solution was intensively mixed for 10 s. A volume of 14 μL of the gel precursor solution was transferred to the surface of a maleimide functionalized PEMA-coated glass coverslips and the gel drop adhering to the coverslip was placed on top of a microstructured PS surface. Next, hydrogels were allowed to form in the presence of the PS microstructures for 2 h at 22 °C. The PS microstructure was dissolved by immersion in xylene (Fluka, Germany). Solvent was exchanged three times after 1 h of incubation. Next the coverslip tethered hydrogels were washed with acetone, ethanol and PBS and left in PBS. Prior to cell culture, the gels were sterilized by washing with a solution of 0.02% ProClin^®^ (Sigma-Aldrich) in PBS.

### Isolation of human CD34+ cells from apharese blood

*Granulocyte colony-stimulating factor* (G-CSF) mobilized human HSPCs were immuno-magnetically isolated for CD34+ cells according to the manufacturer’s instructions (CD34 micro bead Kit UltraPure human, Miltenyi Biotec, Germany). All leukapheresis products were provided by the University Hospital Carl Gustav Carus, Dresden. Leukapheresis and subsequent cell experiments were carried out in accordance to the approved guidelines of the ethics committee of the University Hospital Carl Gustav Carus Dresden including a written informed consent from healthy donors. Briefly, a sample of 2 mL leukapheresis blood was mixed with 4 mL ACK lysing buffer (Invitrogen, Germany) and incubated for 5 min to lyse all red blood cells. Afterwards the sample was centrifuged at 300 g for 5 min (*Sigma 4-16KS*, Sigma Laborzentrifugen GmbH, Germany) and the supernatant was discarded. The remaining cell pellet was washed with 12 mL of isolation buffer (2 mM EDTA and 0.2% HSA in PBS) (both Sigma-Aldrich) and suspended in the staining solution containing 400 μL FcR blocking agent, 400 μL anti-human CD34-antibody (conjugated to 50 nm sized magnetic beads) and 1200 μL isolation buffer. After incubation for 30 min at 4 °C, the samples were washed by the addition of 10 mL isolation buffer and centrifuged at 300 g for 5 min. Subsequently, cells were separated with a MACS^®^ LS column (Miltenyi Biotec) attached to a MACS^®^ separator (Miltenyi Biotec) retaining the magnetically labeled cells within the column. The flow through was discarded and the CD34+ cells were flushed in a 5 mL isolation buffer after removal of the column from the magnetic field of the separator. To increase the purity of CD34+ cells, the procedure was repeated with a MACS^®^ MS column subsequent to the first separation, and the magnetically captured CD34+ cells were flushed in 1.5 mL isolation buffer. Cell numbers and surface marker expression were measured with a MACSQuant Analyzer (Miltenyi Biotec).

### Cell culture and analysis of human HSPCs on top of microcavity arrays

Cells were grown in serum-free CellGro^®^ SCGM medium (CellGenix, Germany) supplemented with 10 or 30 ng·mL^−1^ of the growth factors SCF (Miltenyi Biotec), TPO and FLT3L (both R&D Systems, MN, USA). Prior to cell isolation, all scaffolds were pre-incubated in 48 suspension well plates (Fisher Scientific GmbH) for >12 h in cell culture media without supplemented cytokines at 37 °C. For cell experiments, 500 μL of cell suspensions containing either 30000 or 60000 freshly isolated cells per mL were added on top of the hydrogel scaffolds. Between 2–3 microcavity samples were used per condition. Cells grown on TCP under the same conditions served as the control. After 7 days in culture the supernatants, including non-adherent and adherent cells, were collected from the surfaces. Cells adhering to the FN functionalized microcavity scaffolds were detached using Accutase^®^ (Thermo Electron Cooperation, Australia) (10 min at 37 °C), washed and resuspended. A portion of the harvested cells was used for live/dead cell staining with propidium iodide (PI) (Miltenyi Biotec) and counted with a MACSQuant Analyzer. The remaining cells were stained in PBS/5% (vol/vol) fetal calf serum (FCS; Biochrom, Berlin, Germany) and 2 mM EDTA using CD34-FITC (clone: AC136) and CD133-APC (clone: 293C2) antibodies and cytometrically measured with MACSQuant Analyzer. Dead cells were excluded using PI. Obtained data were analyzed and processed using FlowJo software (Tree Star Inc., OR, USA).

### Secreted factor expression analysis in HSPC cultures

HSPC-secreted factors were measured using multiplex immunoassays based on the xMAP^®^ technology (Luminex, TX, USA). Three different Bio-Plex Pro™ magnetic cytokine, chemokine, and growth factor assays were used: AngioPlex, 27-Plex and the TGF-β 3-Plex assay (BioRad, CA, USA). After 7 days of cell culture, the medium including all cells, was collected and centrifuged at 300 g for 5 min. Equal volumes of cell culture supernatants were stored at −80 °C until analysis. After thawing 110–160 μL aliquots from at least two donors per condition were transferred into a 96 protein low binding plate supported with 0.5% FCS. Samples were transferred in 50 μL volumes into a Bio-Plex Pro™ 96 flat bottom well plate (BioRad) and the assay procedures were performed according to the manufacturer´s instructions. Technical replicates were collected in parallel. Automated washing was performed using a Bio-Plex Pro™ wash station equipped with a magnet (BioRad). Finally, samples were analyzed via cytometric imaging with a Bio-Plex 200 and analyzed with the Bio-Plex Manager™ Software, Standard Edition (both BioRad).

### Partial least squares analysis of the mechanistic model

For partial least square (PLS) analysis of the mechanistic model, the software JMP^®^ Pro (10.0.1.) and the software integrated NIPALS algorithm were used^27^. Obtained HSPC cell numbers were set as the *y* response and the measured growth factor levels, paracrine or autocrine signaling activities, adhesive as well as cell-cell interactions, and heparin content of the scaffolds were set as *x* variables. Values for the *x* variables were parameterized as shown in Fig. 3. Expecting dense hexagonal packing and considering empty and partially filled cavities, cell-cell interactions in the 40 μm microcavity condition were parameterized assuming interactions from 9 of 12 maximal possible cells per cavity. Samples missing the concentration of only one factor *i*, mostly caused by technical reasons, were completed by using the mean value of factor *i* calculated from all samples. Data were centered and scaled to have mean 0 and a standard deviation of 1. The optimum numbers of extracted factors were calculated applying the K-Fold cross validation method. The Van der Voet T^2^ test was applied to test whether models with different numbers of extracted factors differ significantly from the optimum model. Based on the results of the cross validation, the minimal numbers of latent factors were chosen to explain at least 90% of the variation of cumulative *x* and *y* variables and to not differ significantly from the optimum model by Van der Voet T^2^ tests. After determining the necessary number of latent factors, coefficients as well as the variable importance in the projection (VIP), a measure estimating the importance of a variable in a PLS model, for each *x* variable were calculated. Subsequently, variables having a smaller VIP score than 0.8 were deleted from the model^52^. The optimized and VIP validated algorithm was tested to predict the HSPC numbers from the set of measured variables and compared with the counted cell numbers. Predicted and obtained cell numbers were plotted and a linear fit plus the coefficient of determination (R^2^) were calculated to validate the prediction of the algorithm.

### Statistical Analysis

All data were analyzed using GraphPad Prism 6 (GraphPad Software Inc., USA) and JMP^®^ Pro (10.0.1.). Analysis of the statistical significance between two samples was performed either by one-way ANOVA or two-way ANOVA (results obtained from at least two donors) and Tukey-Kramer’s post hoc test. Asterisk denote statistical significance as follows: *P < 0.05, **P < 0.01, ***P < 0.001. All data are presented as mean values ± standard deviation.

## Additional Information

**How to cite this article**: Müller, E. *et al*. Distinguishing autocrine and paracrine signals in hematopoietic stem cell culture using a biofunctional microcavity platform. *Sci. Rep.* **6**, 31951; doi: 10.1038/srep31951 (2016).

## Supplementary Material

Supplementary Information
