# Supplementary material for: Distinguishing autocrine and paracrine signals in hematopoietic stem cell culture using a biofunctional microcavity platform
Source: Sci Rep. 2016 Aug 18;6:31951. doi: 10.1038/srep31951 (PMC4989144; doi:10.1038/srep31951)
Supplement: Supplementary Information [file srep31951-s1.pdf]

Supplementary Information

Distinguishing autocrine and paracrine signals in hematopoietic stem cell culture using a biofunctional microcavity platform

Eike Müller[a,1], Weijia Wang[b], Wendy Qiao[b], Martin Bornhäuser[c], Peter W. Zandstra[b], Carsten Werner[a], Tilo Pompe[a,d,2]

[a] Leibniz Institute of Polymer Research Dresden, Max Bergmann Center of Biomaterials, Dresden, Germany
[b] University of Toronto, Toronto, Canada
[c] TU Dresden, University Hospital Carl Gustav Carus, Dresden, Dresden, Germany
[d] Institute of Biochemistry, Universität Leipzig, Leipzig, Germany

[1] Present address: Laboratory for Biointerfaces, Empa, Swiss Federal Laboratories for Material Science and Technology, CH-9014 St. Gallen, Switzerland

[2] To whom correspondence should be addressed: Prof. Dr. Tilo Pompe, Institute of Biochemistry Universität Leipzig, Johannisallee 21-23, 04103 Leipzig, Germany

phone/fax: +49 (0) 341 9736 931/ 939 ; e-mail: tilo.pompe@uni-leipzig.de

SI Figures

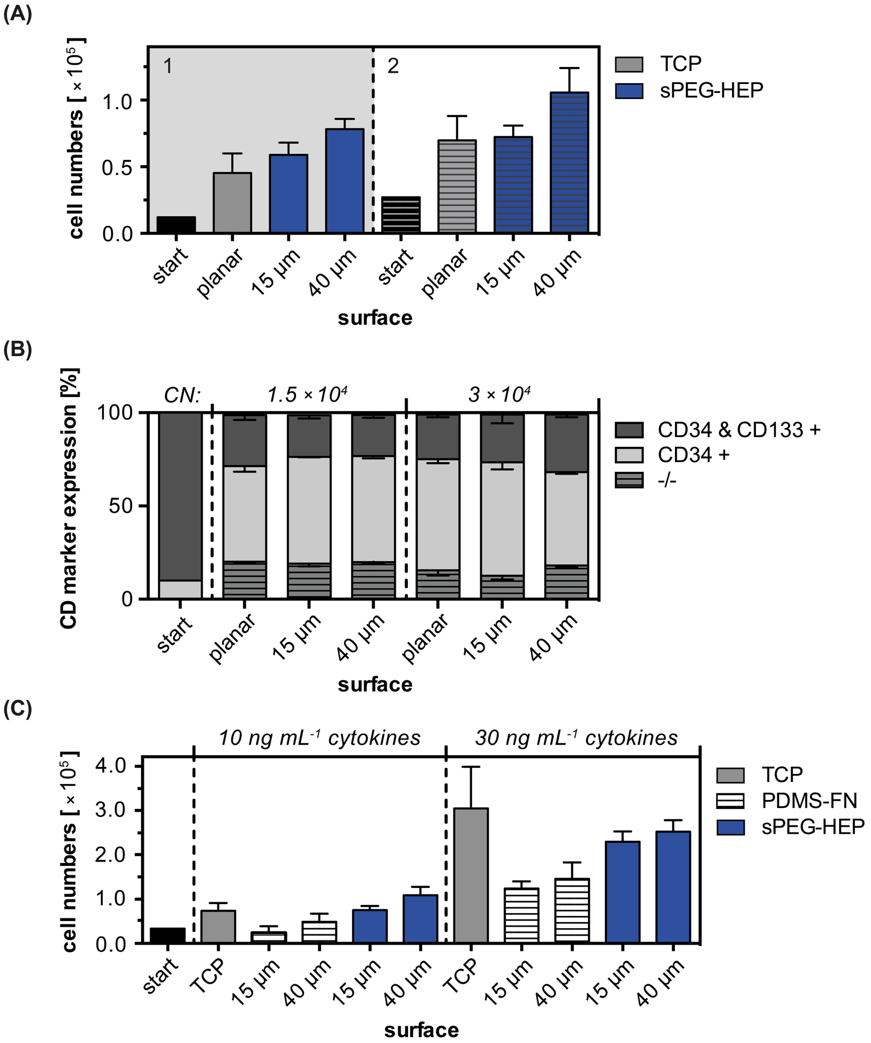

Figure S1. Influence of initial cell numbers and cytokine concentration on the expansion of HSPC grown on microcavity arrays. (A) Expansion of HSPCs cultured for 1 week on top of TCP and hydrogel culture carriers with 15 or 40 µm cavity diameters at 10 ng mL^{-1} cytokine conditions. Initially, 15000 (1) or 30000 (2) cells were seeded per scaffold. (B) Expression of surface stem cell markers CD34 and CD133 was measured by flow cytometry. All data are presented as mean ± SD from one donor and n = 3 surfaces per condition. (C) HSPC number after 7d culture on top of TCP and microcavities made of FN-functionalized PDMS or starPEG-heparin hydrogel scaffolds, at physiological (10 ng mL^{-1}) and increased (30 ng mL^{-1}) cytokine conditions. All data are presented as mean ± SD from one donor and n = 2 - 3 surfaces per donor.

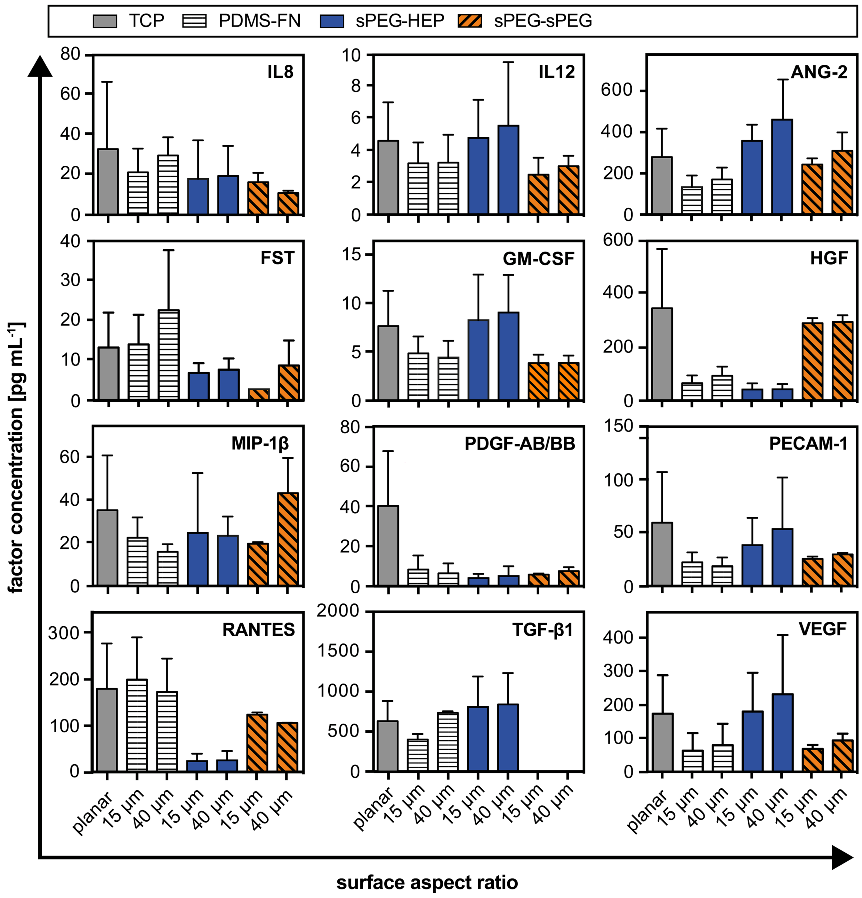

Figure S2. Growth factor concentrations measured in the supernatant after 7d of HSPC culture. 30000 CD34+ HSPCs were cultured on top of PDMS-FN, sPEG-HEP, or sPEG-sPEG microcavity arrays and TCP. The medium was supplemented with 10 ng mL^{-1} cytokines. Supernatant concentrations of secreted factors were measured using multiplex analysis. All data are presented as mean ± SD from 1 (sPEG-sPEG), 2 (PDMS-FN), or 3 (sPEG-HEP) donors and n = 2 - 3 surfaces per condition and donor.

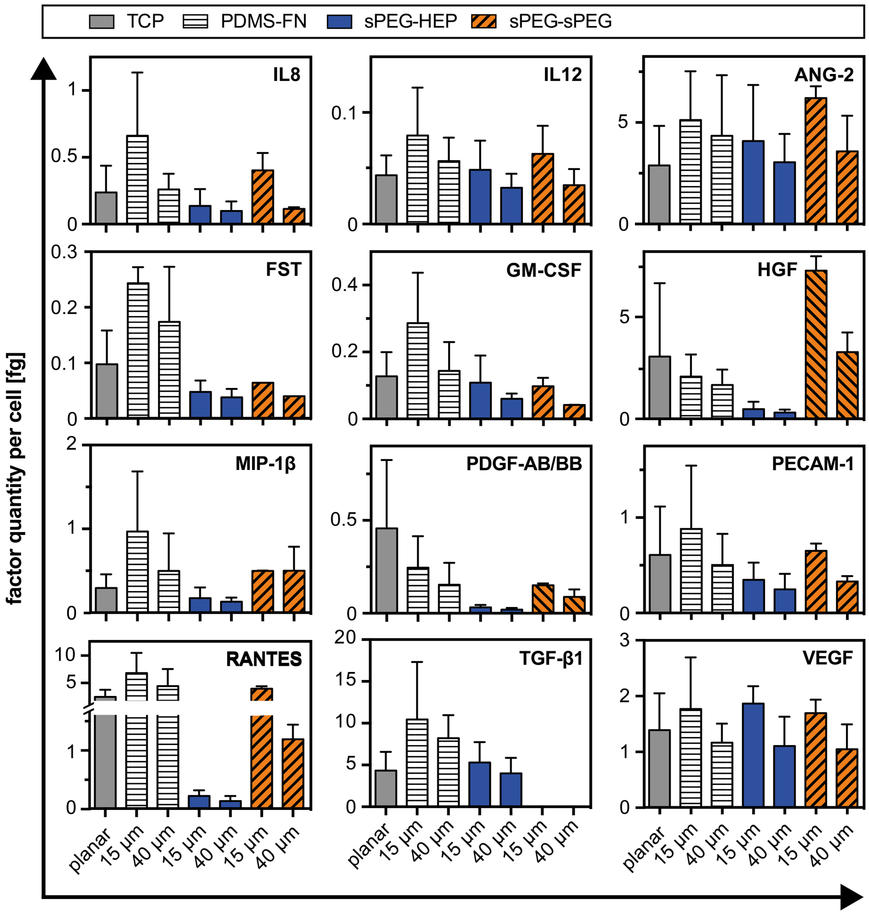

Figure S3. Ratio of growth factor concentrations in the supernatant and final cell numbers after 7d of HSPC culture. 30000 CD34+ HSPCs were cultured on top of TCP, PDMS-FN, sPEG-HEP, or sPEG-sPEG microcavity arrays and the medium supplemented with 10 ng mL^{-1} cytokines. After 7d of culture cell numbers were counted and the supernatant concentrations of secreted factors were measured using multiplex analysis (Fig. S2). Measured growth factor levels were normalized in ratio to the corresponding final cell numbers. All data are presented as mean ± SD from 1 (sPEG-sPEG), 2 (PDMS-FN), or 3 (sPEG-HEP) donors and n = 2 - 3 surfaces per condition and donor.

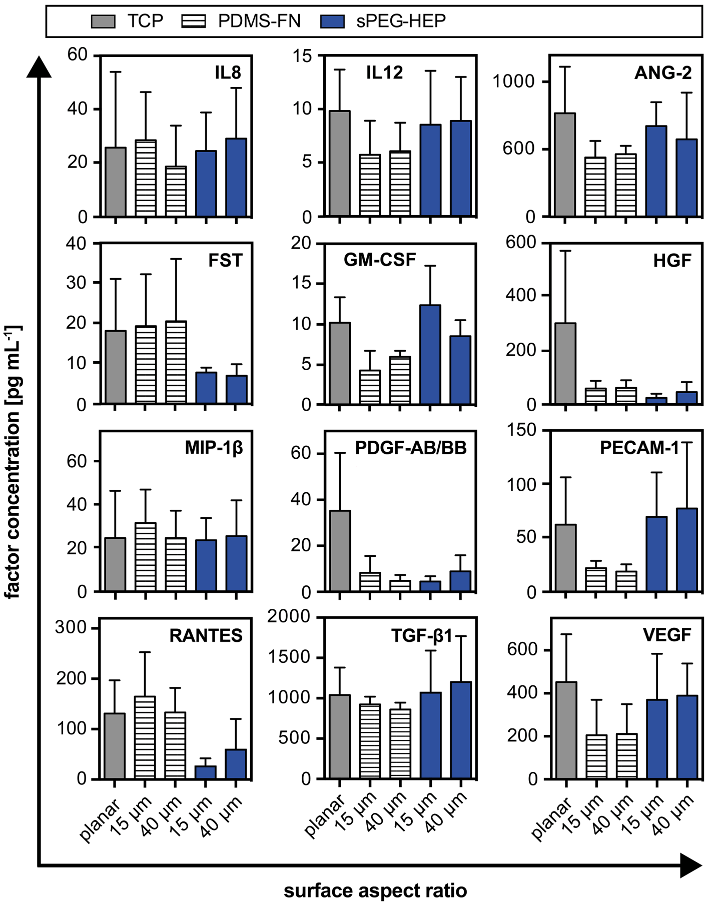

Figure S4. Growth factor concentrations measured in the supernatant after 7d of HSPC culture at increased cytokine concentrations. 30000 CD34+ HSPCs were cultured on top of TCP, PDMS-FN, or sPEG-HEP microcavity arrays and the medium supplemented with 30 ng mL^{-1} cytokines. Obtained HSPC derived factor concentrations in the supernatant were measured using multiplex analysis. All data are presented as mean ± SD from 2 (PDMS-FN) or 3 (sPEG-HEP) donors and n = 2 - 3 surfaces per condition and donor.

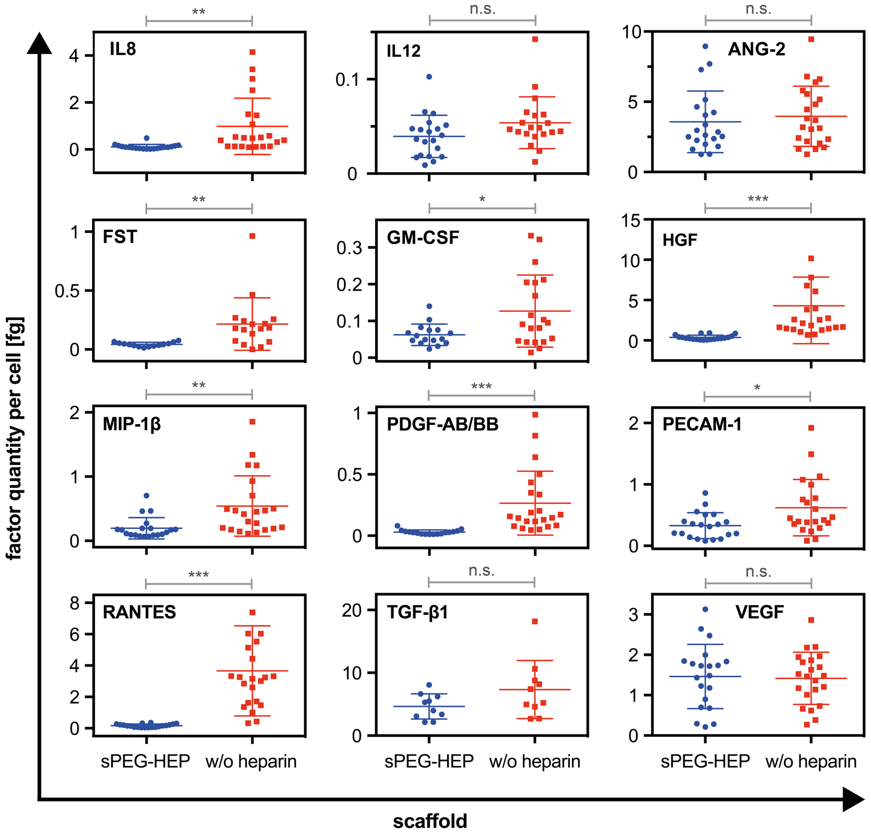

Figure S5. Obtained factor concentrations in the supernatants after 7d of HSPC culture are influenced by the heparin content of the scaffold. 30000 CD34+ HSPCs were cultured on top of TCP, PDMS-FN or hydrogel (sPEG-HEP and sPEG-sPEG) microcavity arrays and the medium supplemented with 10 ng mL^{-1} cytokines. Measured factor concentrations in the supernatant (Fig. S3.) were normalized in ratio to the corresponding final cell numbers. Factor quantity per cell was plotted as function of the heparin content of the scaffold. Statistical two-tailed Student t tests performed through Prism 6 for MacOSX software (*P = 0.05–0.01; **P = 0.01–0.001; ***P < 0.001).
